# Chemical Composition and Biological Activities of Essential Oil from *Salvia sclarea* Plants Regenerated *in vitro*

**DOI:** 10.3390/molecules14041438

**Published:** 2009-04-02

**Authors:** Łukasz Kuźma, Danuta Kalemba, Marek Różalski, Barbara Różalska, Marzena Więckowska-Szakiel, Urszula Krajewska, Halina Wysokińska

**Affiliations:** 1 Department of Biology and Pharmaceutical Botany, Medical University of Łódź, Muszyńskiego 1, 90-151 Łódź, Poland; E-mail: botanika@pharm.am.lodz.pl (H.W.); 2 Institute of General Food Chemistry, Technical University of Łódź, Stefanowskiego 4/10, 90-924 Łódź, Poland; E-mail: dakal@snack.p.lodz.pl (D.K.); 3 Department of Pharmaceutical Biochemistry, Medical University of Łódź, Muszyńskiego 1, 90-151 Łódź, Poland; E-mails: mrozalski@pharm.am.lodz.pl (M.R.), ukrajewska@pharm.am.lodz.pl (U.K.); 4 Department of Immunology and Infectious Biology, Institute of Microbiology and Immunology, University of Łódź, Banacha 12/16, 90-237 Łódź, Poland; E-mail: rozab@biol.uni.lodz.pl (B.R.), mwiec@biol.uni.lodz.pl (M.W-S.)

**Keywords:** *Salvia sclareain vitro* and *in vivo*, Essential oil chemical composition, Antimicrobial and cytotoxic activity.

## Abstract

The essential oils obtained by hydrodistillation of dried aerial parts of *Salvia sclarea* L. plants, regenerated *in vitro* and reproduced from seeds, were analyzed by GC and GC-MS. The oils from *in vitro* and *in vivo* plants were compared in respect to their chemical composition as well as antimicrobial and cytotoxic activities. The chemical profiles of both oils were very similar, although the yield of essential oil from *in vitro* plants was lower (0.1%, v/w) than the oil yield isolated from *in vivo S. sclarea* plants (0.2%, v/w). Both oils showed antimicrobial and cytotoxic activity. The oil from *in vitro* regenerated plants of *S. sclarea* exhibited stronger cytotoxic action against NALM-6 cell lines in comparison with the essential oil from *in vivo* plants.

## Introduction

The genus *Salvia* consists of many species, which have wide applications in folk medicine and also many commercial uses, especially in the production of essential oils and flavoring agents. *Salvia sclarea* L. (clary sage) is an example of such species. This plant, occurring in the Mediterranean basin and Iran [1], is one of the most important aromatic plants cultivated worldwide as a source of essential oils. The essential oils or extracts of the aerial part of the *S. sclarea* plant have a broad spectrum of effects: analgesic, antiinflammatory [2], antioxidant, antifungal [3], and antibacterial [4,5]. Apart from the various medicinal uses, essential oils of clary sage are widely applied in the food and cosmetic industries. 

*In vitro* propagation techniques offer a powerful tool for mass multiplication of plants with a high level of secondary metabolites [6]. In the present study, we report on the yield and chemical composition of the essential oil isolated from *S. sclarea* plants obtained from shoot tip culture. For comparison, the essential oil from clary sage plants propagated from seeds was also isolated and analyzed. The essential oils isolated from *in vitro-* and *in vivo*-derived plants of *S. sclarea* were tested for their cytotoxic and antimicrobial activity. All the plants grew in the field under the same conditions and were collected at the same stage of development. Up to now, we have found no studies on essential oils produced by micropropagated plants of *S. sclarea*. In the literature, there have also been no attempts to investigate the chemical composition of essential oil of *Salvia sclarea* cultivated under Polish climatic conditions, although several studies on the composition of essential oils of the plant species cultivated in many other countries have been published [7,8,9].

## Results and Discussion

The hydrodistillation of the aerial parts of *S. sclarea in vitro* and *in vivo* plants gave yellow oils in 0.1% and 0.2 % (v/w; ml 100 g^-1^ dry weight) yield, respectively. The chemical composition of the essential oils was analyzed by GC and GC-MS. Thus, eighty-two constituents were identified, representing about 96% of the total oils. The compounds, together with their retention indices and relative percentage concentrations are presented in Table 1, according to the elution order on the CP Sil 5 CB column. It was found that the chemical profiles of both oils were similar. The oils comprised 21 oxygenated monoterpenes (74% and 75% in the oils from *in vitro* and *in vivo* plants, respectively), 13 monoterpene hydrocarbons (8.8%; 7.8%), 19 sesquiterpene hydrocarbons (6.6%; 4.3%), 18 oxygenated sesquiterpenes (5.8%; 6.4%), and 7 oxygenated diterpenes (1.1%; 2.2%) (Table 1). The principal components of both oils were identified as linalool (42.3% in the oil from *in vitro* plants and 38.6% in the oil from *in vivo* plants), α-terpineol (13.4%; 14.3%), geraniol (6.3%; 7.7%), its acetate derivative (5.4%; 5.8%), and myrcene (3.3%; 3.4%). Linalool is one of the most useful monoterpene alcohols for the perfumery industry as well as for synthesis route to vitamin E [10]. Furthermore, this compound has exhibited antinociceptive [11], anticonvulsant [12] and sedative activities [13].

A major difference between the oils from *in vitro* and *in vivo S. sclarea* plants was observed only in the content of germacrene D; in the oil from micropropagated plants the amount of the sesquiterpene was almost four times higher than that found in the oil from *in vivo* plants. Similarity in the chemical composition of essential oils from *in vitro* and *in vivo* plants has been reported by some other authors [14,15]. For example, Fortunato and Avato (2008) [14] have reported that a chemical profile of the essential oil from *Origanum vulgare* L. ssp. *hirtum* *in vitro* plants was comparable to that of the control mother plants, with carvacrol as the main compound. On the other hand, comparative studies on the essential oils from *in vitro* and *in vivo* plants of *Salvia przewalskii* showed numerous differences between the two oil profiles [16]. We have found that the samples of oils studied by us were different from samples of oils obtained from *S. sclarea* plants grown for example in Greece, Spain, or Yugoslavia [7,8]. According to Souleles and Argyriadou (1997) [7], in the oil isolated from *S. sclarea* grown in Greece, 72 constituents were identified. Linalool was the main constituent of this oil, but its content (17.2%) was over twice lower than that detected in essential oils isolated in the present study. In contrast, linalyl acetate was found in the essential oil from Greek clary sage in a higher amount (14.3%) [7] as compared to the samples of oils analyzed in the present work (1.1-2.6%). In the essential oil from aerial parts of *S. sclarea* growing in Yugoslavia [8], the percentage content of geraniol (1.2%) was significantly lower than that in*in vitro* and *in vivo* clary sage plants examined in our work (6.3%; 7.7%). The sample of oil from plants growing in Spain showed an increased content of germacrene D (7.6%) [7] in comparison with oils analyzed in our laboratory (Table 1). The changes in composition of oils could be related to climate or soil conditions.

The oils from *in vitro* and *in vivo* plants of *S. sclarea* were evaluated for their cytotoxicity and antimicrobial activity (Table 2 and Table 3). Among the bacteria tested, *Escherichia coli* strain was the most sensitive microorganism to clary sage essential oils (Table 2). However, the significant activity was also noted against *S. aureus* and *S. epidermidis* strains (MIC values, respectively 10.0 and 5.0 mg mL^-1^). The antimicrobial activity of the essential oils obtained from studied *S. sclarea,* propagated *in vitro* or *in vivo,* may well be due to the presence of synergy, antagonism or additive effects of the tested major components of the oils, which possess various potency of activity. Further studies are needed to explain which essential oils component, out of 82 main constituents demonstrated, are the most important one and is responsible for the bactericidal effects observed in this study.

As shown in Table 3 both oil samples tested exhibited cytotoxic activity against the NALM-6 and HL-60 cell lines, although the promyelocytic leukemia HL-60 cells were more sensitive to the essential oils (IC_50_ = 6.5 µg/mL). The essential oil isolated from *in vitro* regenerated plants of *S. sclarea* had over 2-fold stronger cytotoxic activity against the NALM-6 cell line (IC_50_ = 8.1 µg/mL) than the volatile oil obtained from *in vivo* clary sage plants (IC_50_ = 20.1 µg/mL). This difference was statistically significant with *p* value less than 0.01, as calculated by Student’s *t*-test. Higher cytotoxic activity could be explained with a higher content of germacrene D in the oil isolated from *in vitro* plants (Table 1). Antiproliferative activity of the sesquiterpene against the MDA-MB-231, MCF7, Hs 578T, PC-3 and Hep-G2 cell lines has been reported by Setzer et al. (2006) [17]. Germacrene D has exhibited a 7-fold stronger cytotoxic activity against the Hs 578T cell line in comparison with α-pinene and limonene and a 6-fold stronger action against Hs 578T and Hep-G2 than 1,8-cineole, linalool, 4- terpineol and α-terpineol [17]. 

**Table 1:** Constituents of essential oils from *in vitro*/*in vivo* *Salvia* *sclarea* plants.

Peak number	Compound	RI	V (%)	S (%)
1.	α-thujene	926	0.1	t
2.	α-pinene	935	t	t
3.	camphene	950	0.1	0.1
4.	β-pinene	974	0.2	t
5.	myrcene	983	3.3	3.4
6.	α-phellandrene	999	0.1	0.1
7.	α-terpinene	1012	t	t
8.	p-cymene	1116	0.1	0.1
9.	limonene	1025	1.0	0.9
10.	(*Z*)-β-ocimene	1031	1.0	0.9
11.	(*E*)-β-ocimene	1041	2.1	1.8
12.	γ-terpinene	1055	0.2	0.1
13.	*trans*-sabinene hydrate	1060	t	t
14.	*trans*-linalool oxide	1062	t	t
15.	fenchone	1070	0.1	0.1
16.	*cis*-linalool oxide	1076	t	t
17.	terpinolene	1083	0.6	0.4
18.	**linalool**	1087	42.3	38.6
19.	*endo*-fenchol	1100	0.2	0.2
20.	camphor	1126	t	t
21.	pinocamphone	1139	0.1	0.1
22.	borneol	1155	0.1	t
23.	terpinen-4-ol	1166	0.2	0.1
24.	**α** **-terpineol**	1178	13.4	14.3
25.	myrtenol	1178	t	t
26.	p-menth-1-en-9-al	1188	t	t
27.	β-cyclocitral	1195	t	t
28.	nerol	1213	2.3	2.5
29.	piperitone	1232	t	t
30.	**geraniol**	1238	6.3	7.7
31.	linalyl acetate	1242	1.1	2.6
32.	neryl formate	1268	0.1	t
33.	bicycloelemene	1339	0.2	0.1
34.	neryl acetate	1344	2.8	3.0
35.	α-cubebene	1355	0.1	0.1
36.	(*E*)-β-damascenone	1362	t	t
37.	**geranyl acetate**	1363	5.4	5.8
38.	α-copaene	1379	1.3	1.2
39.	β-bourbonene	1386	0.2	0.2
40.	β-elemene	1390	0.3	0.1
41.	α-cedrene	1418	0.1	0.1
42.	β-caryophyllene	1420	1.1	1.1
43.	geranylacetone	1430	0.1	t
44.	calarene	1437	t	t
45.	isogermacrene D	1445	0.1	t
46.	(*Z*)-β-farnesene	1448	t	t
47.	α-humulene	1453	0.1	0.1
48.	(*E*)-β-farnesene	1475	t	t
49.	β-ionone	1477	0.2	0.2
50.	germacrene D	1480	2.2	0.6
51.	β-selinene	1484	t	0.1
52.	bicyclogermacrene	1493	0.3	0.1
53.	cubebol	1514	0.1	0.1
54.	*trans*-calamenene	1514	t	t
55.	δ-cadinene	1516	0.3	0.2
56.	α-calacorene	1527	0.2	0.2
57.	salviadienol	1540	0.1	0.2
58.	β-calacorene	1541	0.1	0.1
59.	mintoxide	1545	t	t
60.	spathulenol	1569	1.5	1.1
61.	caryophyllene oxide	1573	1.4	2.2
62.	salvial-4(14)en-1-one	1584	0.4	0.3
63.	humulene epoxide II	1587	0.1	0.1
64.	torilenol	1601	0.1	0.1
65.	cedrol	1602	0.2	0.2
66.	12-*epi*-cedrol	1620	0.1	0.1
67.	humulene epoxide III	1624	0.2	0.2
68.	T-cadinol	1633	0.2	0.1
69.	β-eudesmol	1641	0.5	0.6
70.	α-eudesmol	1651	0.3	0.3
71.	eudesma-4(15),7-dien-1β-ol	1671	0.3	0.4
72.	α-bisabolol	1673	0.1	0.1
73.	(*Z,Z*)-farnesol	1685	0.1	0.2
74.	(*E,E*)-farnesol	1706	0.1	0.1
75.	(*E*)-2,6-dimethyl-10-(p-tolyl)-undeca-2,6-diene	1945	0.2	0.3
76.	manoyl oxide	2007	0.4	0.6
77.	13-*epi*-manoyl oxide	2023	0.2	0.2
78.	manool	2070	0.1	0.3
79.	13-*epi*-manool	2080	0.2	0.4
80.	labda-7,14-dien-13-ol	2096	0.1	0.1
81.	isoabienol	2124	t	t
82.	sclareol	2231	0.1	0.6
	**Oxygenated monoterpenes**		**74.4**	**75.0**
	**Monoterpene hydrocarbons**		**8.8**	**7.8**
	**Sesquiterpene hydrocarbons **		**6.6**	**4.3**
	**Oxygenated sesquiterpenes **		**5.8**	**6.4**
	**Oxygenated diterpenes **		**1.1**	**2.2**
	**Other components **		**0.5**	**0.5**
	**Total**		**97.2 **	**96.2**

RI - Relative retention index on CP Sil 5 CB column; V - essential oil from *in vitro* plants; S - essential oil from *in vivo* plants; t - trace (percentage value less than 0.01%).

**Table 2:** Antimicrobial activity of *S. sclarea* essential oils. MIC values were determined by the microdilution assay, according to CLSI recommendations.

Microorganism	MIC		
	[mg mL^-1^]		[µg mL^-1^]
	V	S	Control
*Staphylococcus aureus* (ATCC 29213)	>5.0	10.0	1.0 (A)
*Staphylococcus epidermidis* (ATCC 12228)	>5.0	5.0	8.0 (A)
*Enterococcus faecalis* (ATCC 29212)	>5.0	>10.0	1.0 (A)
*Escherichia coli* (NCTC 8196)	>5.0	2.5	0.015 (O)
*Pseudomonas aeruginosa* (NCTC 6749)	>5.0	>10.0	2.0 (O)
*Candida albicans* (ATCC 10231)	>5.0	>10.0	8.0 (I)

MIC - Minimum inhibitory concentration; V - *In vitro* plants; S - *In vivo* plants; Control antimicrobial agents: (A) - ampicillin; (O) – ofloxacin; (I) – itraconazole

**Table 3:** Cytotoxic activity of *S. sclarea* essential oils. IC_50_ values were determined by the MTT assay.

Essential oil	HL-60	NALM-6
	IC_50_ [µg mL^-1^]	
V	6.5 ± 0.3	8.1 ± 0.7
S	6.4 ± 0.4	20.1 ± 3.4

**IC_50_ - **The concentration of the tested oil required to reduce the cell survival fraction to 50% of the control; V - *In vitro* plants; S - *In vivo* plants

## Conclusions

Our studies have shown that the yield of the oil isolated from *in vitro* plants of *S. sclarea* at the flowering stage was slightly lower to that of *in vivo* plants. However, the chemical profile and relative amounts of compounds were very similar in both oils. These results strongly suggest that using *in vitro* propagated plants of *S. sclarea* as a source of essential oil is possible. It is important in view of the biological activity and commercial application of the essential oil.

## Experimental

### Plant material

According to the reported procedure [18], shoots of *Salvia sclarea* were multiplied from shoot tips on solid (0.7% agar) Murashige and Skoog (MS) medium [19] supplemented with indole-3-acetic acid (IAA) (0.1 mg L^-1^), 6-benzylaminopurine (BAP) (1.0 mg L^-1^) and sucrose (3%). For rooting, axillary shoots were transferred on agar MS medium without growth regulators. Cultures were maintained in the growth room at 26 ± 2 ºC and 70% humidity with photoperiod of 16 h of light/8 h of darkness. Illumination was supplied by cool white fluorescent lamps with a light intensity of 40µmol m^-2^ s^-1^. Four-week-old rooted shoots were transferred to the pots with the sterile mixture of soil, sand and peat (4:3:3) and grown under greenhouse conditions, at 24 ºC. After acclimatization stage (3 weeks), plantlets were grown for two years in the field (Medicinal Plant Garden of the Medical University of Łódź). They were referred to as *in vitro* plants. For comparison, *Salvia sclarea* plants propagated from seeds and cultivated under the same conditions as *in vitro* plants were also investigated. They were referred to as *in vivo* plants. For analysis, *S. sclarea* plants *in vitro* and *in vivo* were two years old. Aerial parts of the plants were collected at the flowering stage. 

### Isolation of essential oil

The essential oil was obtained by hydrodistillation of the air-dried aerial parts of *in vitro* and *in vivo* plant materials (shoots and flowers) (*ca* 140 g dry weight) using a Clevenger-type apparatus for 5 h.

### GC and GC-MS analyses

The GC analysis of the essential oil samples was performed using a Carlo-Erba Vega 6000 apparatus equipped with an FID detector and a capillary column CP Sil 5 CB (30 m × 0.32 mm i.d., film thickness 0.25 µm, Quadrex Corporation, New Haven). The oven temperature was programmed as follows: 60 – 300 ºC at 4 ºC/min and the final temperature was held for 10 min; injector temperature 320 ºC; detector temperature 310 ºC; carrier gas N_2_ at a flow rate of 1.5 ml min^-1^. Peaks were measured by electronic integration. The percentage composition of the essential oil samples was computed from the peak areas using the normalization method. The GC-MS analysis was carried out with a Fison MD 800 mass spectrometer (ion source 200 ºC, EI 70 eV) connected to a GC 8000 gas chromatograph; helium served as the carrier gas with a flow rate of 0.8 mL min^-1^. A CP Sil 5 CB column was used at the same parameters as described for GC. The identification of compounds was based on the comparison of their retention indices and mass spectra with those in commercial libraries NIST 98.1 and MassFinder 3.1. The results are presented in Table 1.

### Biological activity of essential oils

For antibacterial and antifungal activity testing, three Gram-positive bacteria: *Staphylococcus aureus* ATCC 29213, *Staphylococcus epidermidis* ATCC 12228, *Enterococcus faecalis* ATCC 29212; two Gram-negative bacteria: *Escherichia coli* NCTC 8196, *Pseudomonas aeruginosa* NCTC 6749 and yeast *Candida albicans* ATCC 10231 were used. The susceptibility of microorganisms to essential oils was determined by the standard CLSI (Clinical Laboratory Standards Institute) microdilution method. Sterile stock solutions of each oil at the concentration of 160 mg mL^-1^ were prepared in dimethyl sulphoxide (DMSO). The oils concentration range used was 10.0-0.15 mg mL^-1^, prepared for bacteria in Mueller-Hinton broth (Difco), and for yeast in RPMI-1640 medium supplemented with L-glutamine and NaHCO_3_ (Biomed, Poland). Overnight grown bacterial suspensions were standarized to 10^6^ CFU mL^-1^ (*E. faecalis* - 10^7^ CFU mL^-1^); the yeast suspension was standarized to 10^5^ CFU mL^-1^. One hundred µL of each suspension was added to the wells containing essential oils serial dilutions (in triplicate). The oils solubilizer was used as a growth control (no antimicrobial activity was noted). The culture medium was used as a blank control. The following standard antibacterial/antifungal agents were used: ampicillin (Serva), ofloxacin (Sigma), itraconazole (Jansen-Cilag Int., N.V.). After incubation at 37^0 ^C, 24 h (bacteria) and at 28^0^C, 48 h (yeast), turbidometric (OD_600_) measures were carried out using the multifunction counter Victor2 (Wallac, Finland) and the minimal inhibitory concentrations (MICs) were specified. The results are shown in Table 2.

### Cytotoxic activity of essential oils

Cytotoxic activity of *S. sclarea* essential oils was determined by the MTT [3-(4,5-dimethylthiazol-2-yl)-2,5-diphenyltetrazolium bromide; Sigma, St. Louis, USA] assay [20]. Human leukemia promyelocytic HL-60 and lymphoblastic NALM-6 cell lines were used. More details about the cell lines and methods of cytotoxicity assay have been described previously by Różalski et al. (2006) [21]. The results are shown in Table 3.
